# Idecabtagene vicleucel for relapsed and refractory multiple myeloma: post hoc 18-month follow-up of a phase 1 trial

**DOI:** 10.1038/s41591-023-02496-0

**Published:** 2023-08-17

**Authors:** Yi Lin, Noopur S. Raje, Jesús G. Berdeja, David S. Siegel, Sundar Jagannath, Deepu Madduri, Michaela Liedtke, Jacalyn Rosenblatt, Marcela V. Maus, Monica Massaro, Fabio Petrocca, Ashish Yeri, Olivia Finney, Andrea Caia, Zhihong Yang, Nathan Martin, Timothy B. Campbell, Julie Rytlewski, Jaymes Fuller, Kristen Hege, Nikhil C. Munshi, James N. Kochenderfer

**Affiliations:** 1 Mayo Clinic, Rochester, MN USA; 2 Massachusetts General Hospital Cancer Center and Harvard Medical School, Boston, MA USA; 3 Sarah Cannon Research Institute and Tennessee Oncology, Nashville, TN USA; 4 Hackensack University Medical Center, Hackensack, NJ USA; 5 Mount Sinai Medical Center, New York City, NY USA; 6 Stanford University Medical Center, Palo Alto, CA USA; 7 Beth Israel Deaconess Medical Center, Boston, MA USA; 8 2SeventyBio, Inc, Cambridge, MA USA; 9 Bristol Myers Squibb, Princeton, NJ USA; 10 Dana-Farber Cancer Institute, Boston, MA USA; 11 Surgery Branch, National Cancer Institute/National Institutes of Health, Bethesda, MD USA

**Keywords:** Targeted therapies, Translational research

## Abstract

Idecabtagene vicleucel (ide-cel) is a B-cell-maturation antigen (BCMA)-directed chimeric antigen receptor T cell therapy. We performed a post hoc analysis of a single-arm phase 1 multicenter study in relapsed/refractory multiple myeloma (CRB-401) (*n* = 62; median follow-up, 18.1 months). The primary endpoint was safety outcomes, and secondary endpoints included overall response rate (ORR), complete response (CR) and very good partial response (VGPR). The study met its primary endpoint with low rates of grade 3/grade 4 cytokine release syndrome (6.5%) and neurotoxicity (1.6%). ORR was 75.8%; 64.5% achieved VGPR or better and 38.7% achieved CR or stringent CR. Among exploratory endpoints, median duration of response, progression-free survival (PFS) and overall survival were 10.3, 8.8 and 34.2 months, respectively, and ide-cel expansion in blood and bone marrow correlated with clinical efficacy and postinfusion reduction of soluble BCMA. Patients with PFS ≥ 18 months had more naive and less exhausted T cells in apheresis material and improved functional T cell phenotype in the drug product compared with those with less durable responses. These results confirm ide-cel safety, tolerability and efficacy and describe T cell qualities that correlate with durable response. Clinicaltrials.gov identifier : NCT02658929.

## Abstract (web summary)

This is a post hoc 18-month follow-up analysis of the CRB-401 trial, testing idecabtagene vicleucel (ide-cel, bb2121) in relapsed and refractory multiple myeloma, and reports sustained safety and clinical efficacy, which correlates with T cell phenotypes.

## Main

Multiple myeloma (MM) is an incurable plasma cell neoplasm associated with substantial morbidity and mortality and generally shorter durations of response with each subsequent line of therapy^1–3^. Although treatment advances have prolonged survival, deep and durable responses are difficult to obtain in the relapsed and refractory setting, with worsening prognosis and survival following each relapse^4^. Patients who are triple-class refractory (that is, previously refractory to the following three commonly used drug classes: immunomodulatory drugs (IMiDs), anti-CD38 monoclonal antibodies and proteasome inhibitors) have poor outcomes with subsequent regimens^5–7^, with a median progression-free survival (PFS) of 2–6 months and a median overall survival (OS) of <12 months^6^, highlighting the need for new treatment approaches.

Autologous anti-CD19 chimeric antigen receptor (CAR) T cell therapy has led to durable clinical remission in many patients with refractory hematologic malignancies^8–10^, and key learnings from this field have broadened this treatment to other disease indications. Favorable intrinsic T cell characteristics, such as lower expression of exhaustion markers, lower glycolytic metabolism, less apoptosis, and higher expression of stem cell memory, central memory and naive markers^11,12^, are correlated with improved clinical responses. Similarly, autologous drug products (DPs) manufactured from patient T cells that display increased potency, better functional activity and lower cell surface expression of differentiation markers were correlated with tumor response and clinical efficacy in chronic lymphocytic leukemia^11^. Whether these intrinsic features of T cell quality correlate with improved clinical responses in autologous CAR T cell therapies outside of those that target CD19 is an important question and impacts future strategies to improve their efficacy.

B-cell maturation antigen (BCMA) is a member of the tumor necrosis factor receptor superfamily that has a key role in the proliferation, maturation and differentiation of B cells^13,14^. BCMA is nearly universally expressed on myeloma and normal plasma cells, with restricted minimal expression in other normal tissue^13,15,16^. Therefore, BCMA is a promising target antigen for the treatment of MM. Idecabtagene vicleucel (ide-cel, bb2121) is a BCMA-directed CAR T cell therapy incorporating an anti-BCMA single-chain variable fragment; a CD8 hinge and transmembrane domain; the 4-1BB costimulatory motif for increased proliferation, expansion and persistence; and a CD3ζ signaling domain^17,18^. In the dose escalation (*n* = 21) and initial dose expansion (*n* = 12) of the phase 1 CRB-401 study, ide-cel demonstrated tolerability and promising efficacy in heavily pretreated patients with relapsed and/or refractory multiple myeloma (RRMM)^19^. Following the publication of that report, enrollment has been completed, and additional 29 patients have been treated in dose expansion. Here we report post hoc safety, efficacy and translational results for all 62 patients who received ide-cel in this ongoing CRB-401 study, which has one of the longest follow-up periods of any multicenter, US-based, anti-BCMA CAR T cell therapy clinical trial. In contrast to recent publications^20,21^, the long follow-up of this trial enabled the identification and reporting of translational correlates of long-term responses following this therapy, including peripheral and bone marrow biomarkers, starting material (peripheral blood (PB) mononuclear cells (PBMCs)) and DP correlates.

## Results

### Patient disposition and characteristics

A total of 67 patients were enrolled and underwent leukapheresis. The first patient underwent leukapheresis on January 14, 2016, and the first ide-cel infusion was administered on February 16, 2016. The last patient had the first ide-cel infusion on January 7, 2019. The data cutoff was April 7, 2020. The manufacturing of ide-cel was successful for 100% of patients. Sixty-two of the enrolled patients underwent lymphodepletion and ide-cel infusion (Supplementary Fig. 1). Five patients discontinued before lymphodepletion due to adverse events (AEs), progressive disease or withdrawal (*n* = 1 each) or physician decision (*n* = 2). As reported previously^19^, 21 patients were enrolled and treated in the dose-escalation phase (3, 6, 9 and 3 patients at 50, 150, 450 and 800 × 10^6^ CAR^+^ T cell doses, respectively). Based on cumulative clinical (including safety, efficacy and translational) data at completion of the dose escalation phase, 150–450 × 10^6^ CAR^+^ T cell dose levels were selected for further expansion. An additional 41 patients were treated in dose expansion, with 12 and 29 treated at 150 and 450 × 10^6^ CAR^+^ T cell doses, respectively.

In the full ide-cel-treated population (*n* = 62), the median age was 61 years, and 43.5% of patients had a high tumor burden (≥50% bone marrow CD138^+^ plasma cells; Table 1). The median number of prior regimens was 6 (range = 3–18), with 45.2% of patients receiving >6 prior regimens. Overall, 93.5% of patients were triple-class exposed, and 69.4% were triple-class refractory. All patients in the dose-expansion cohort were triple-class exposed, and 82.9% were triple-class refractory (Supplementary Table 1). Furthermore, patients in the dose-expansion cohort were older relative to the dose-escalation cohort (median age, 63 and 57 years, respectively), and a greater proportion had Eastern Cooperative Oncology Group performance status (ECOG PS) of 1 (80.5% and 52.4%, respectively) and extramedullary disease (46.3% and 19.0%, respectively). A greater proportion of patients in the dose-expansion cohort compared with the dose-escalation cohort (58.5% and 38.1%, respectively) received bridging therapy administered between leukapheresis and ide-cel infusion.

**Table 1:** Baseline characteristics and prior treatments

Characteristics	50 × 10^6^ CAR^+^ T cells (*n* = 3)	150 × 10^6^ CAR^+^ T cells (*n* = 18)	450 × 10^6^ CAR^+^ T cells (*n* = 38)	800 × 10^6^ CAR^+^ T cells (*n* = 3)	Total (*n* = 62)
Age, median (range), years	60 (58–68)	63.5 (44–75)	61 (37–74)	57 (41–67)	61 (37–75)
Male, *n* (%)^a^	2 (66.7)	13 (72.2)	23 (60.5)	1 (33.3)	39 (62.9)
Time since diagnosis, median (range), years	1.5 (1.4–6.2)	6.2 (1.7–15.2)	5.4 (0.8–35.7)	4.1 (3.9–15.9)	5.5 (0.8–35.7)
ECOG PS 0/1, %^b^					
0	1 (33.3)	5 (27.8)	9 (23.7)	1 (33.3)	16 (25.8)
1	1 (33.3)	13 (72.2)	28 (73.7)	2 (66.7)	44 (71.0)
High-risk cytogenetics, *n* (%)^c^	0	6 (33.3)	10 (26.3)	1 (33.3)	17 (27.4)
R-ISS III at baseline, *n* (%)	1 (33.3)	2 (11.1)	7 (18.4)	1 (33.3)	11 (17.7)
High tumor burden, *n* (%)^d^	2 (66.7)	10 (55.6)	14 (36.8)	1 (33.3)	27 (43.5)
Extramedullary disease, *n* (%)	2 (66.7)	4 (22.2)	16 (42.1)	1 (33.3)	23 (37.1)
No. of prior regimens, median (range)	4 (3–11)	8 (4–15)	6 (3–18)	6 (5–7)	6 (3–18)
Prior ASCT, *n* (%)	3 (100)	16 (88.9)	35 (92.1)	3 (100)	57 (91.9)
Prior therapies, exposed/refractory, %					
Last prior therapy	100/33.3	100/61.1	100/89.5	100/33.3	100/75.8
Bortezomib	100/33.3	100/77.8	94.7/52.6	100/33.3	96.8/58.1
Carfilzomib	66.7/0	100/55.6	89.5/68.4	100/100	91.9/62.9
Ixazomib	0/0	16.7/16.7	36.8/28.9	33.3/33.3	29.0/24.2
Lenalidomide	100/33.3	100/83.3	100/71.1	100/66.7	100/72.6
Pomalidomide	33.3/0	100/77.8	94.7/89.5	100/33.3	93.5/79.0
Daratumumab	33.3/0	94.4/77.8	92.1/86.8	100/33.3	90.3/77.4
Isatuximab	0/0	11.1/11.1	7.9/7.9	0/0	8.1/8.1
Immunomodulatory and PI	100/33.3	100/83.3	100/84.2	100/66.7	100/80.6
Immunomodulatory, PI and anti-CD38	33.3/0	94.4/72.2	97.4/76.3	100/33.3	93.5/69.4
Bridging therapy, *n* (%)	1 (33.3)	9 (50.0)	21 (55.3)	1 (33.3)	32 (51.6)

^a^Data on sex were not collected in this study.

^b^Two patients (1 each at 50 and 450 × 10^6^ target cells) had ECOG PS 2.

^c^del17p, *t*(4;14) and/or *t*(14;16).

^d^≥50% CD138-positive cells or percentage of plasma cells in bone marrow biopsy. ASCT, autologous stem cell transplant; PI, proteasome inhibitor.

The median follow-up for all surviving (censored) patients with ongoing survival was 18.1 (range = 1.5–44.5) months, and 11 of 62 patients had ongoing responses (2, 8 and 1 at 150, 450 and 800 × 10^6^ CAR^+^ T cell doses, respectively). Reasons for discontinuation were progressive disease (*n* = 38; 61.3%), patient withdrawal (*n* = 6; 9.7%), death (*n* = 6; 9.7%) and other (*n* = 1; 1.6%).

### Primary outcome: safety

All patients experienced an AE of any grade (Table 2), and all but 1 patient experienced a grade 3/grade 4 AE. The most common grade 3 /grade 4 AEs were hematologic in nature—neutropenia (88.7%), leukopenia (61.3%), anemia (56.5%) and thrombocytopenia (56.5%). The median times to recovery from grade 3/grade 4 to grade ≤2 neutropenia and thrombocytopenia (in patients with persistent cytopenia 1 month after ide-cel infusion) were 1.9 (range = 1.2–3.0) months and 2.2 (range = 1.1–8.7) months, respectively.

**Table 2:** AEs of special interest, absolute number (%)

AEs	Any grade	Grade 3/grade 4
Any	62 (100)	61 (98.4)
Neutropenia	57 (91.9)	55 (88.7)
Febrile neutropenia	10 (16.1)	8 (12.9)
Anemia	47 (75.8)	35 (56.5)
Infection^a^	47 (75.8)	14 (22.6)
CRS^b^	47 (75.8)	4 (6.5)
Thrombocytopenia	46 (74.2)	35 (56.5)
Leukopenia	40 (64.5)	38 (61.3)
Lymphopenia	23 (37.1)	22 (35.5)
Neurotoxicity^c^	22 (35.5)	1 (1.6)
Second primary malignancy	8 (12.9)	2 (3.2)

^a^Includes the SOC infections and infestations.

^b^CRS uniformly graded per ref. ^30^.

^c^Grouped term; events reported ≤8 weeks after infusion. Excludes 1 patient with grade 1 insomnia lasting 251 d.

Any-grade cytokine release syndrome (CRS) was reported in 47 patients (75.8%). These events were predominantly grade 1/grade 2 (*n* = 43; 69.4%); 4 patients (6.5%) had a grade 3 event and none reported a grade ≥4 event (Table 3). The incidence of any-grade CRS generally increased with the target dose level, with 2 of 3 patients (67%) at 50 × 10^6^, 7 of 18 (38.9%) at 150 × 10^6^, 35 of 38 (92.1%) at 450 × 10^6^ and 3 of 3 (100%) at 800 × 10^6^ CAR^+^ T cells. The median time of onset was 2 d, and the median duration was 5 d. Tocilizumab and corticosteroids were used in 29.0% and 16.1% of patients, respectively. No differences in CRS event or grade were noted between dose-expansion and dose-escalation cohorts. There were no reports of macrophage activation syndrome or hemophagocytic lymphohistiocytosis.

**Table 3:** CRS and neurotoxicity

	50 × 10^6^ CAR^+^ T cells (*n* = 3)	150 × 10^6^ CAR^+^ T cells (*n* = 18)	450 × 10^6^ CAR^+^ T cells (*n* = 38)	800 × 10^6^ CAR^+^ T cells (*n* = 3)	Total (*n* = 62)
CRS^a^					
Any grade, *n* (%)	2 (66.7)	7 (38.9)	35 (92.1)	3 (100.0)	47 (75.8)
1	2 (66.7)	3 (16.7)	20 (52.6)	1 (33.3)	26 (41.9)
2	0	4 (22.2)	12 (31.6)	1 (33.3)	17 (27.4)
3	0	0	3 (7.9)	1 (33.3)	4 (6.5)
4	0	0	0	0	0
Time to first onset, median (range), days	10 (1–19)	2 (1–17)	2 (1–11)	1 (1–4)	2 (1–19)
Duration, median (range), days	2 (1–3)	4 (1–22)	5 (1–32)	4.5 (3–7)	5 (1–32)
Tocilizumab use, *n* (%)	0	2 (11.1)	14 (36.8)	2 (66.7)	18 (29.0)
Corticosteroid use, *n* (%)	0	1 (5.6)	9 (23.7)	0	10 (16.1)
Neurotoxicity^b^					
Any grade, *n* (%)	0	3 (16.7)	19 (50.0)	1 (33.3)	23 (37.1)
1	0	3 (16.7)	14 (36.8)	1 (33.3)	18 (29.0)
2	0	0	4 (10.5)	0	4 (6.5)
3	0	0	0	0	0
4	0	0	1 (2.6)	0	1 (1.6)
Time to first onset, median (range), days	–	10 (5–22)	5 (1–30)	4 (4–4)	5 (1–30)
Duration, median (range), days	–	4 (4–4)	3 (1–62)	2 (2–2)	3 (1–62)
Corticosteroid use, *n* (%)	0	1 (5.6)	4 (10.5)	0	5 (8.1)

^a^CRS uniformly graded per ref. ^30^.

^b^Grouped term; events reported ≤8 weeks after infusion. Only 1 patient had a neurotoxicity event after 8 weeks that was possibly related to ide-cel (grade 1 tremor on day 61). Excludes 1 patient treated with 150 × 10^6^ CAR^+^ T cells who had grade 1 insomnia lasting 251 d that was not suspected to be related to ide-cel.

Any-grade neurotoxicity (grouped term) within 8 weeks of infusion was reported in 23 patients (37.1%) and was primarily grade 1/grade 2 (*n* = 22; 35.5%), with 1 patient (1.6%) who received 450 × 10^6^ CAR T cells experiencing a grade 4 event of reversible neurotoxicity with cerebral edema (beginning on day 11 and lasting until day 42). No occurrences of parkinsonism or Guillain–Barre syndrome were reported. Similar to CRS rates, the rate of neurotoxicity increased with increasing target dose levels as follows: no patients at 50 × 10^6^, 16.7% of patients at 150 × 10^6^, 50.0% at 450 × 10^6^ and 33.3% at 800 × 10^6^ CAR^+^ T cells (Table 3). The median time of onset was 5 d after infusion, and events lasted a median of 3 d. Corticosteroids were used in 5 (8.1%) patients. Neurotoxicity was similar between dose-escalation and dose-expansion cohorts.

Any-grade infections were reported in 75.8% of patients (including 22.6% with grade 3/grade 4 events). Eight patients had second primary malignancies reported after ide-cel infusion (myelodysplastic syndromes (MDS) and Bowen disease in 2 patients each; breast cancer, malignant melanoma, adenocarcinoma of the colon, basal cell carcinoma and bladder cancer occurred in 1 patient each (the latter two events occurred in the same patient)). Both patients who developed MDS during the study had previously received alkylating agents and had cytogenetic abnormalities (5q and 7q deletions detected at the time of diagnosis) that are known to be typical of MDS associated with alkylating agent chemotherapy.

One patient died within 8 weeks of ide-cel infusion. The patient who experienced grade 2 CRS events on days 1 and 8 (which resolved on days 4 and 12, respectively) achieved minimal response on day 31, withdrew from care and died in hospice of unknown cause 51 d after receiving ide-cel at a target dose of 150 × 10^6^ CAR^+^ T cells. Seven additional patients died within 6 months (1 due to cardiopulmonary arrest not attributable to ide-cel and 6 due to MM progression).

### Secondary outcomes

**Fig. 1:**
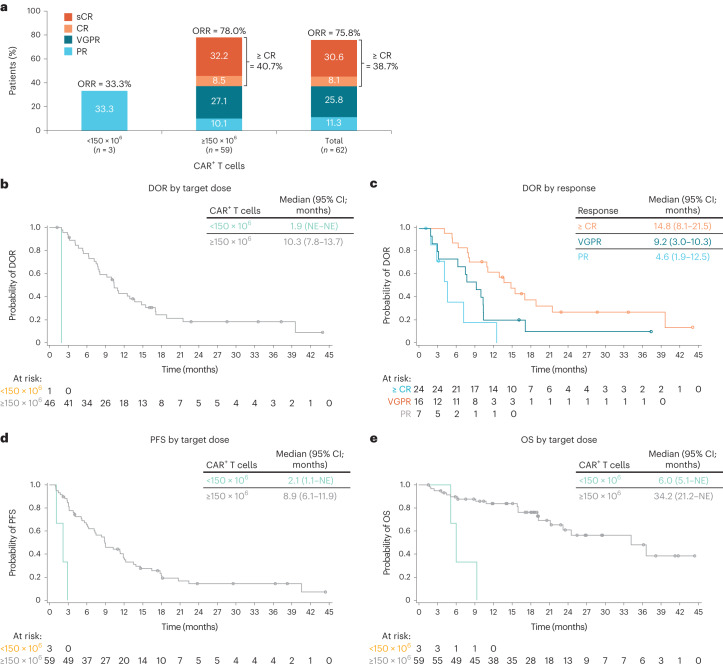
Efficacy outcomes in all dose groups. Efficacy outcomes include the following: **a**–**e**, Best overall response by dose (**a**), DOR by dose (**b**), DOR by best overall response (**c**), PFS by dose (**d**) and OS by dose (**e**).

The overall response rate (ORR) in all patients was 75.8%, with 64.5% achieving a very good partial response (VGPR) or better and 38.7% achieving a complete response (CR) or stringent CR (sCR; Fig. 1a and Supplementary Fig. 2a). Response rate and depth increased with higher ide-cel dose, with an ORR of 33.3% at 50 × 10^6^, 50.0% at 150 × 10^6^, 89.5% at 450 × 10^6^ and 100.0% at 800 × 10^6^ CAR^+^ T cells (≥VGPR, 0.0%, 38.9%, 36.8% and 100.0%, respectively). Response rates differed between dose-escalation and dose-expansion cohorts (Supplementary Table 2), likely due (at least in part) to differences in baseline patient characteristics between the two cohorts or to the low number of patients in each treatment group (Supplementary Table 1).

### Exploratory outcomes

Responses were durable, with a median duration of response (DOR) in all patients of 10.3 months. The median DORs were 1.9 (95% confidence interval (CI), not estimable (NE)–NE) months, 13.7 (95% CI = 2.9–NE) months, 10.0 (95% CI = 6.3–14.8) months and 12.9 (20.9–NE) months in patients treated with 50, 150, 450 and 800 × 10^6^ CAR^+^ T cells, respectively (Fig. 1b and Supplementary Fig. 2b). Of note, 4 of the 8 patients with ongoing response had responses lasting >2 years, all of whom received doses ≥150 × 10^6^ CAR^+^ T cells (*n* = 1, 150 × 10^6^; *n* = 2, 450 × 10^6^; *n* = 1, 800 × 10^6^ CAR^+^ T cells), and the longest reported DOR was >40 months. Increased depth of response was associated with longer median DOR. The median DOR was 4.6 (95% CI = 1.9–12.5) months in the 7 patients with partial response (PR), 9.2 (95% CI = 3.0–10.3) months in the 16 patients who achieved VGPR and 14.8 (95% CI = 8.1–21.5) months in the 24 patients with CR or sCR (Fig. 1c). Durable responses were also observed across multiple subgroups treated with 150–450 × 10^6^ CAR^+^ T cells, including older patients, those with higher Revised International Staging System (R-ISS) stage or extramedullary disease, and those who received bridging therapy and treatment with 150–450 × 10^6^ CAR^+^ T cells (Supplementary Fig. 3).

At the time of data cutoff (median follow-up, 18 months), median PFS across all dose groups was 8.8 (95% CI = 5.9–11.9) months and median OS was 34.2 (95% CI = 19.2–NE) months. Kaplan–Meier estimates of OS indicate that 79.2% and 57.7% of patients across all dose groups were alive at 1 year and 2 years, respectively. PFS and OS outcomes were dose-dependent (Fig. 1d,e and Supplementary Fig. 2c,d). Survival outcomes were numerically shorter (median PFS, 2.1 (95% CI = 1.1–NE) months; median OS, 6.0 (95% CI = 5.1–NE) months) in the 3 patients treated at target doses of <150 × 10^6^ compared with the 59 patients treated with ≥150 × 10^6^ CAR^+^ T cells (median PFS, 8.9 (95% CI = 6.1–11.9) months; median OS, 34.2 (95% CI = 21.2–NE) months).

Fourteen of 15 patients (93.3%) who achieved ≥CR and underwent an assessment for bone marrow minimal residual disease (MRD) within 3 months before CR achieved MRD-negative status (10^–5^ sensitivity; Supplementary Fig. 4). Of the remaining 9 patients who achieved ≥CR, 8 did not have an assessment for MRD and 1 had an assessment outside of the 3-month window before ≥CR. None of the patients had MRD-positive assessments as their only evidence of residual myeloma.

### Correlates of durable response

**Fig. 2:**
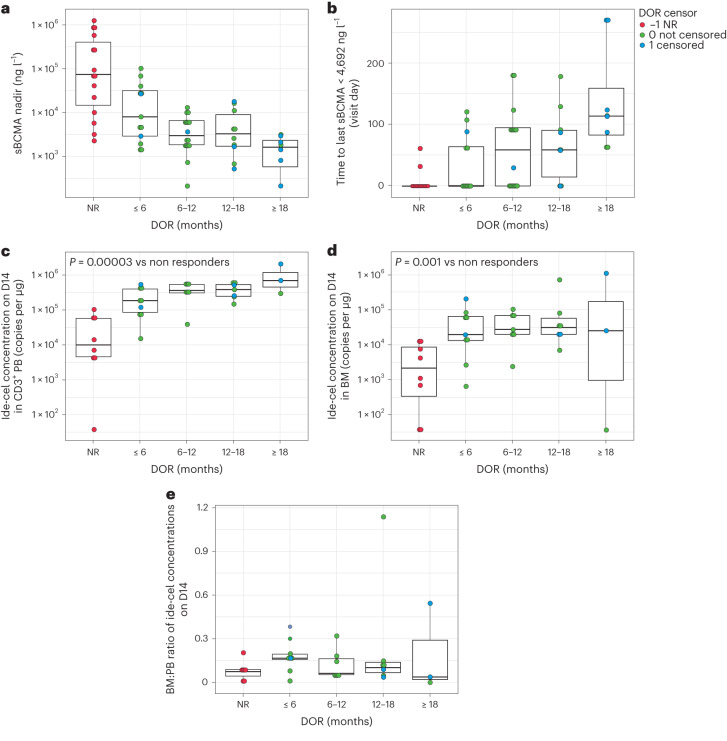
sBCMA dynamics and cellular kinetics associated with DOR. Box and whisker plots are provided, whereby the center horizontal line denotes the median, and the box denotes the IQR (25th–75th percentiles). The upper whisker extends to the maximum value, but no further than 1.5× IQR above the 75th percentile, and the lower whisker extends to the minimum value, but no lower than 1.5× IQR below the 25th percentile. Data beyond 1.5× IQR from the 25th or 75th percentiles, respectively, are plotted individually as outlying points. **a**, sBCMA nadir by DOR: >18 months, *n* = 8 patients; 12–18 months, *n* = 10 patients; 6–12 months, *n* = 16 patients; <6 months, *n* = 13 patients; NR, *n* = 15 patients. **b**, Duration of deep pharmacodynamic (greater sBCMA reduction) response by DOR: >18 months, *n* = 8 patients; 12–18 months, *n* = 10 patients; 6–12 months, *n* = 16 patients; <6 months, *n* = 13 patients; NR, *n* = 15 patients. **c**, Concentrations, measured by qPCR, of ide-cel in the PB CD3^+^ cell matrix on day 14 postinfusion by DOR (*P* = 0.00003 versus nonresponders, two-sided Wilcoxon rank-sum test; *P* value is not corrected for multiple hypothesis testing): >18 months, *n* = 3; 12–18 months, *n* = 7; 6–12 months, *n* = 7; <6 months, *n* = 9; NR, *n* = 8. **d**, Concentrations, measured by qPCR, of ide-cel in bone marrow aspirate (whole bone marrow matrix) on day 14 postinfusion by DOR (*P* = 0.001 versus nonresponders, two-sided Wilcoxon rank-sum test; *P* value is not corrected for multiple hypothesis testing): >18 months, *n* = 3; 12–18 months, *n* = 7; 6–12 months, *n* = 7; <6 months, *n* = 9; NR, *n* = 8. **e**, Ratio of bone marrow aspirate (whole bone marrow matrix) to PB CD3^+^ cell matrix concentrations of ide-cel on day 14 postinfusion by DOR. DOR was binned as 0–6, 6–12, 12–18 and ≥18 months. Duration of deep pharmacodynamic response was defined by the last visit at which sBCMA levels were less than the median nadir (4692 ng l^−1^): >18 months, *n* = 3; 12–18 months, *n* = 7; 6–12 months, *n* = 7; <6 months, *n* = 9; NR, *n* = 8. BM, bone marrow; IQR, interquartile range; NR, nonresponder.

The concentration of BCMA in serum, a peripherally accessible and composite marker of change in myeloma tumor burden, was significantly greater in patients with myeloma who had progressive disease as best overall response compared with those achieving a response of PR or better to ide-cel, and changes in levels of circulating soluble BCMA (sBCMA) in serum were correlated with changes in an individual patient’s clinical status^15^. The nadir of sBCMA levels after ide-cel infusion was lower in responding patients, and there was a numerical trend toward increasing depth of sBCMA clearance in the peripheral blood (PB) with longer DOR; however, no clear trend was seen between median sBCMA nadir and best overall response (Fig. 2a). Additionally, as expected, a greater duration of deeper sBCMA reduction was associated with increasing DOR. Notably, patients with DOR > 18 months maintained sBCMA levels below the median nadir value (that is, 4692 ng l^−1^) for the longest duration (Fig. 2b), demonstrating an ongoing low or absent myeloma burden. No patients had very low or undetectable levels of sBCMA at the time of progression. One patient who had sBCMA levels around the median nadir level, consistent with expression levels in normal plasma cells, at the time of relapse was subsequently shown to have a loss of BCMA within tumor cells^22^.

This phase 1 trial included paired sampling of bone marrow and PB at day 14 postinfusion, enabling the determination of CAR T cell concentration (by vector transgene copies) in both compartments. Expanding on previous findings that focused on PB measurements^19^, greater CAR T cell expansion was observed in both the PB and bone marrow compartments in responding patients compared with nonresponding patients (Fig. 2c,d). However, the ratio of bone marrow to PB CAR T cell concentrations was similar in responding and nonresponding patients and was independent of DOR (Fig. 2e). This observation suggests that overall CAR T cell expansion, rather than an ability to home to marrow alone, drove clinical response.

### Correlates of durable response

**Fig. 3:**
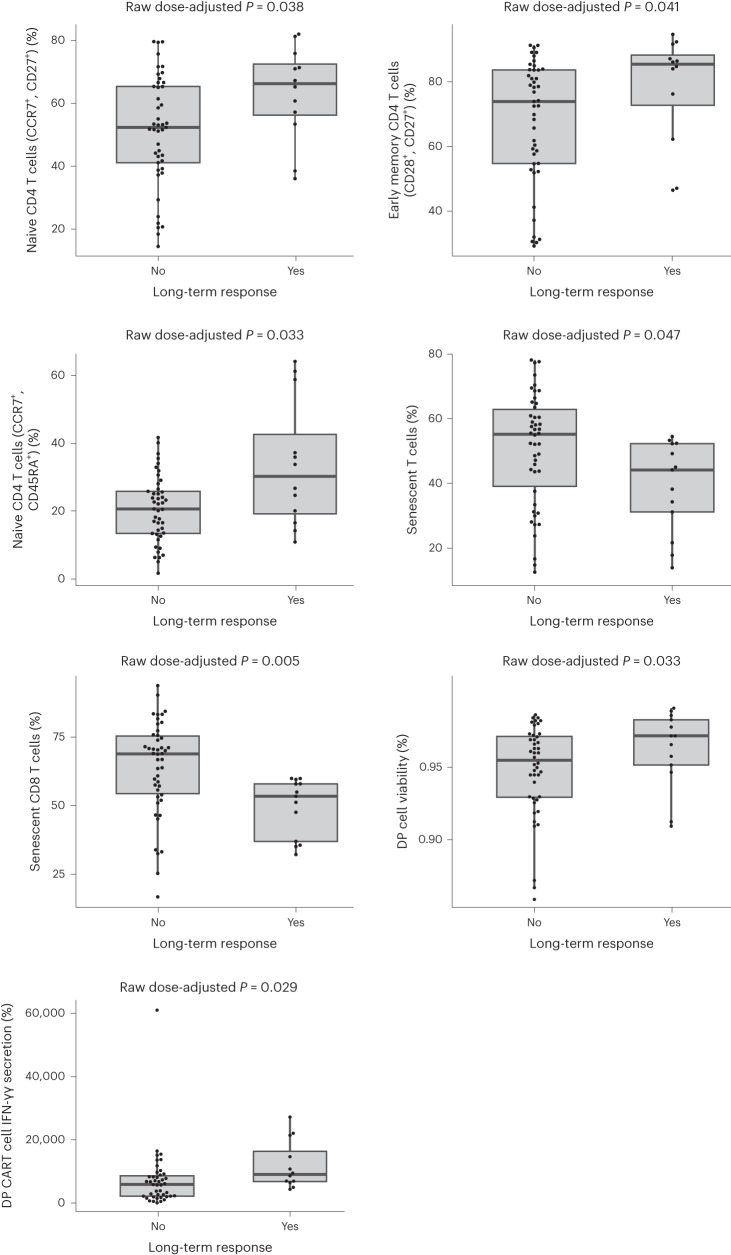
Correlates of long-term responders. Logistic regression analyses of starting material PBMC and final DP correlates from long-term responders (patients with PFS ≥ 18 months; *n* = 13) and non-long-term responders (*n* = 46) and time of recent exposure to selected prior antimyeloma drug classes as a potential correlate of the PBMC and DP variables enriched in long-term responders. Three patients were censored from the total treated population (*n* = 62) due to study discontinuation before progression (*n* = 1), start of subsequent antimyeloma therapy before progression (*n* = 1) and ongoing response with a <18-month progression-free interval (*n* = 1). Note that the *y*-axis labels corresponding to the staining patterns defined for each type of cell are shown in the table of median values below the plots. Box plots show nominal *P v*alue of the long-term response coefficient from a nonparametric linear regression where rank (*Y*) ~ long-term response + dose and *Y* is the rank order value of the PBMC or DP attribute of interest*. P* values are not corrected for multiple hypothesis testing.

Thirteen of the 62 treated patients (21%) achieved a PFS interval of ≥18 months. These results were used to identify apheresis PBMC and DP correlates of long-term response to ide-cel. T cell phenotyping via standard flow cytometry was performed on both the PBMC starting material from apheresis and DP to evaluate T cell memory subsets, phenotypes associated with senescence and T cell dysfunction, and anti-BCMA-stimulated CAR T cell activity via interferon (IFN)-γ secretion of the DP. Statistically significant correlates of long-term response included a higher percentage of naive and early memory (CD28^+^ and CD27^+^) CD4 T cells, naive (CCR7^+^ and CD45RA) and a lower percentage of senescent (CD57^+^) CD3 and CD8 T cells in PBMC starting material for manufacturing (Fig. 3), suggesting that improved intrinsic T cell quality (less-differentiated, more-proliferative cells) in PBMC was favorable for a durable ide-cel response. Correlates of long-term response in ide-cel DP included higher target antigen-specific IFN-γ secretion and cell viability, suggesting more robust effector functionality on stimulation (Fig. 3).

Recent exposure to various prior antimyeloma therapies (hematopoietic stem cell transplantation (HSCT), alkylators, anti-CD38 antibody, corticosteroids, IMiDs and proteasome inhibitors) was analyzed as a potential correlate of the PBMC features enriched in long-term responders (Fig. 3). More recent exposure to alkylators or HSCT was associated with a higher percentage of senescent CD8 T cells in the PBMCs, while recent exposure to IMiDs was associated with a lower percentage of senescent CD8 T cells. Recent HSCT was also associated with fewer naive CD4 T cells in the PBMCs.

## Discussion

Patients with RRMM with triple-class exposure have poor outcomes and a high unmet need for new therapies^5–7^. This post hoc analysis expands upon the first reported results of phase 1 CRB-401 study^19^ by increasing enrollment to 41 patients in the dose-expansion cohort (from 12 in the first report) and lengthening the median follow-up to 18 months (from ≈11 months in the first report). The inclusion criteria were designed to select a population that was more refractory to standard antimyeloma drug classes (particularly triple class) compared with the dose-escalation group. This report presents the long-term OS in these patients and conducts exploratory analyses of PBMC and DP characteristics and durability of treatment response. Overall, ide-cel demonstrated deep and durable dose-dependent responses, with several patients achieving responses lasting >2 years and with a median OS approaching 3 years.

CRS and neurotoxicity are common events with CAR T cell treatments. In CRB-401, there were relatively few grade 3 CRS events (*n* = 4; 6.5%) and no grade 4 events. Similarly, neurotoxicity was most often grade 1/grade 2, with no grade 3 and 1 reversible grade 4 (1.6%) event; no new grade 3/grade 4 neurotoxicity events were reported in the 41 additional patients who received ide-cel reported here. Clinical practice has evolved to include earlier use of tocilizumab and steroids to manage CRS and neurotoxicities. Thus, it is notable that a low incidence of severe CRS and neurotoxicities was observed here because a minority of patients received tocilizumab or steroids for the management of these toxicities. Cytopenias were common but generally acute, reversible and manageable. Grade 3/4 infections were reported in 22.6% of patients. While infections are common in patients with RRMM, the lymphodepleting conditioning regimen and cytopenias may have contributed to this baseline infection risk. Of the eight second primary malignancies, only one (MDS) was considered related to ide-cel. Molecular analyses in both cases of MDS demonstrated cytogenetic abnormalities consistent with the use of prior alkylating agent therapies.

Based upon the totality of the efficacy and safety results during the dose escalation phase, the safety monitoring committee supported the recommended phase 2 dose range of 150–450 × 10^6^ CAR^+^ T cells for the dose expansion study. This range provided durable activity and manageable safety with relatively few grade 3/grade 4 CRS and neurotoxicity events. Indeed, this target dose range has been studied in the pivotal phase 2 KarMMa study (*n* = 128), with results generally consistent with those from CRB-401 (median follow-up, ≈13 months)^21^.

One observation from the CRB-401 study was that the median OS (34.2 months) was much longer than the median PFS (8.9 months). There are several possible explanations for this difference, including that CAR T therapy in most patients provides a prolonged period without any antimyeloma therapy, which may allow better tolerance of subsequent therapies postrelapse and outcomes. Recent real-world data on outcomes postrelapse following anti-BCMA CAR T therapy support this possible explanation^23,24^.

Relative to the first reported data from CRB-401 (ref. ^19^), the larger number of patients treated in this study, the longer length of median follow-up and the larger complementary translational data set allowed for new exploratory analyses of potential biomarkers with the goal of further elucidating key correlates of durable response to ide-cel. These correlates focused on the patients with the most durable responses among the 62 patients treated, and included the manufacturing PBMC starting material and final DP T cell phenotype, CAR T cell expansion and longitudinal changes in sBCMA. Consistent with previous reports from the CRB-401 and KarMMa clinical trials^19,21^ and similar to what has been reported in CD19 CAR T cell studies^25–27^, postinfusion CAR T cell expansion remains a key determinant of response, and both the depth and duration of sBCMA reduction were associated with improved DOR. Elevated sBCMA levels were observed in almost all patients at the time of relapse; however, genomic BCMA loss was subsequently demonstrated in 1 patient^22^, underscoring that this may be a potential, but likely rare, relapse mechanism.

Key correlates of long-term response to ide-cel included more naive, less-differentiated T cells and fewer senescent cells in the PBMC manufacturing starting material. Recent exposure to select antimyeloma therapies such as HSCT, IMiDs and alkylators likely had an impact on the PBMC features associated with long-term response, with IMiD exposure being associated with generally favorable responses and HSCT/alkylators associated with less favorable responses. The finding that durable response is correlated with more highly functional DP CAR T cells is consistent with findings reported in CD19 CAR T cell studies^28^. Overall, these findings suggest that favorable immune health leading to improved T cell intrinsic quality, postinfusion memory and persistence may be important in autologous cell therapy outcomes, independent of any specific tumor antigen, disease indication or manufacturing process^25^.

The current analyses of the CRB-401 study had a few potential limitations. First, because this was a phase 1 study, differences in survival endpoints such as PFS and OS in patients treated with ide-cel could not be fully elucidated because these endpoints were exploratory. Despite this limitation, prior studies of CAR T cell therapies also reported large differences in PFS and OS similar to those observed in the CRB-401 trial of axicabtagene ciloleucel in patients with refractory large B-cell lymphoma (for example, PFS, 5.8 months; OS, not reached with a median follow-up of 15.4 months, with an estimated 24-month OS of 50.5% (refs. ^26,29^)), suggesting that PFS and OS differences may reflect the unique biology of CAR T therapy. In addition, the overall patient enrollment for this phase 1 study was relatively small, and analyses for long-term endpoints were made with data from a small number of patients (for example, OS at 24 months, *n* = 13). This limited statistical power for exploratory assessments (for example, biomarkers and subgroup analyses), the results of which await validation in larger trials. Finally, patients enrolled in this study were highly refractory to prior therapies and had more advanced disease; thus, these results may not apply to the early stages of the disease, in which ide-cel is being studied in ongoing clinical trials.

Overall, the long-term results from CRB-401 continue to demonstrate the safety and tolerability of ide-cel. Further, deep and durable long-term responses were observed with ide-cel in heavily pretreated patients with RRMM, with a favorable clinical benefit-risk profile at target dose levels ≥150 × 10^6^ CAR^+^ T cells. Translational correlates of durable response to ide-cel have been identified that can further inform the field of autologous CAR T cell therapy, with the goal of selecting the patient population with characteristics that are favorable for the most durable responses in patients treated with these therapies. The activity of ide-cel is being further explored in trials currently underway in earlier-line myeloma treatment settings (KarMMa-2, NCT03601078; KarMMa-3, NCT03651128; KarMMa-4, NCT04196491; and KarMMa-7, NCT04855136).

## Methods

### Study design

CRB-401 (NCT02658929) is an open-label, two-part, phase 1 study of ide-cel dose escalation and dose expansion. The current analysis is a post hoc, 18-month follow-up of this phase 1 study. As described previously in an interim analysis^19^, a 3 + 3 dose-escalation approach was used in part 1. The primary endpoint was safety (the incidence of dose-limiting toxicities and maximum tolerated dose in part 1 and confirmation of safety in part 2 (dose expansion)). Secondary endpoints were ORR, CR rate, VGPR and PR according to International Myeloma Working Group (IMWG) criteria^31^. Exploratory endpoints included OS, PFS, MRD, quantification of ide-cel over time and quantification of circulating sBCMA over time. After disease progression, patients were asked to participate in a separate long-term (up to 15 years) follow-up study. The study was conducted in accordance with the general ethical principles outlined in the Declaration of Helsinki. Institutional review boards approved the protocol at each study center, and each patient provided written informed consent.

### Patients

Patients with RRMM who were ≥18 years of age and had ECOG PS 0-1 were eligible for the study. Patients were required to have measurable disease (serum M-protein ≥0.5 g dl^−1^, urine M-protein ≥200 mg 24 h^−1^, or involved free light chain ≥10 mg dl^−1^ with abnormal serum free light chain ratio). Before a protocol amendment in May 2018, patients with measurable disease as indicated by bone marrow plasma cell percentage or extramedullary plasmacytomas alone were also eligible. In dose escalation, patients had to have received ≥3 different prior lines of therapy, including a proteasome inhibitor (for example, bortezomib or carfilzomib) and IMiD (for example, lenalidomide or pomalidomide), or have disease that was double refractory to a proteasome inhibitor and IMiD. Patients in the dose-escalation group were also required to have evidence of BCMA expression in ≥50% of malignant plasma cells (bone marrow biopsy or plasmacytoma) via validated immunohistochemistry of formalin-fixed, paraffin-embedded tumor tissue. The dose-expansion part of the study enrolled patients who had received ≥3 prior lines of therapy, including a proteasome inhibitor, an IMiD and the anti-CD38 antibody daratumumab, and were refractory (per IMWG criteria^31^) to their last line of therapy. Patients in the dose-expansion group could have BCMA expression in <50% of malignant plasma cells. Exclusion criteria for both dose escalation and dose expansion included known central nervous system disease or a history of clinically relevant central nervous system pathology. Additional criteria were published previously^19^.

### Treatment

PMBCs obtained from leukapheresis were stimulated with αCD3 and αCD28 antibodies, transduced with the anti-BCMA CAR-containing lentiviral vector and expanded over 10 d as previously described^17^. Bridging therapy was permitted up until 14 d before lymphodepletion. Patients received one 3-d cycle of lymphodepletion (fludarabine, 30 mg m^−2^ d^−1^ and cyclophosphamide, 300 mg m^−2^ d^−1^) on days −5, −4 and −3. Following 2 d of rest, patients received an infusion of ide-cel on day 0 at target doses of 50, 150, 450 and 800 × 10^6^ total CAR^+^ T cells in dose escalation and target doses of 150–450 × 10^6^ total CAR^+^ T cells in dose expansion. One patient who received 205 × 10^6^ CAR^+^ T cells and one patient who received 305 × 10^6^ are included under the 450 × 10^6^ target dose.

### Assessments

The IMWG Uniform Response Criteria for Multiple Myeloma were used to assess response and progression^31^. AEs were graded according to the National Cancer Institute Common Terminology Criteria for Adverse Events version 4.03. CRS grading and management followed published guidance^30^. Neurotoxicity was reported ≤8 weeks after infusion as a grouped term (comprising preferred terms including, but not limited to, bradyphrenia, brain edema, confusional state, hallucination, insomnia, lethargy, memory impairment, neurotoxicity, nystagmus, somnolence and tremor). MRD negativity was assayed via next-generation sequencing (clonoSEQ, Adaptive Biotechnologies; threshold, 10^−4^ nucleated cells). Time to cytopenia recovery was determined via univariate statistics without adjustment for censoring. The median survival follow-up is the conventional median of time from infusion to the last known date alive for all surviving patients. Other statistical analyses were performed as reported previously^19^.

### Translational

Cellular kinetics (PB and bone marrow) and sBCMA were evaluated as previously described^19^. Cellular kinetics were evaluated via quantitative polymerase chain reaction (qPCR), and results were analyzed for associations with response and DOR in patients with matched day 14 samples in CD3^+^ cells sorted by flow cytometry from PB and in whole bone marrow aspirate. sBCMA was measured in serum via enzyme-linked immunosorbent assay or Luminex and was evaluated for each patient at the time points available; the nadir was the minimum value observed for postinfusion time points. The duration of deep pharmacodynamic responses (sBCMA response) was determined as the time from infusion to the last visit day on which sBCMA was below the median nadir (4692 ng l^−1^). Patients without a nadir <4692 ng l^−1^ were defined as having 0 d of deep pharmacodynamic sBCMA response.

### PBMC and product characterization

PBMC used for ide-cel manufacturing and DP characteristics were evaluated from cryopreserved samples produced during the ide-cel clinical manufacturing process. PBMC or DP samples were thawed, washed and stained with cocktails of fluorescently labeled antibodies targeting T cell differentiation, exhaustion and senescence markers as described below. Flow cytometry was performed to assess phenotypic characteristics of PBMC and DP T cells based on surface expression of CD45RA (BD Biosciences, HI100-BV605; volume per test = 5, final staining concentration = 1.25 µg ml^−1^), CCR7 (BD Biosciences, 150503-BV421; volume per test = 5, final staining concentration = 2.5 µg ml^−1^), CD28 (Thermo Fisher Scientific, CD28.2-PE eFlour610; volume per test = 20, final staining concentration = 2.5 µg ml^−1^) and CD27 (BD Biosciences, L128-FITC; volume per test = 10, final staining concentration = 0.08 µg ml^−1^; T cell differentiation), and expression of senescent and T cell dysfunction-associated markers such as CD57 (BD Biosciences, NK-1-BB515; volume per test = 5, final staining concentration = 1.875 µg ml^−1^), TIM-3 (Thermo Fisher Scientific, F38-2E2-Super Bright 600; volume per test = 5, final staining concentration = 1.25 µg ml^−1^), LAG-3 (BioLegend, 11C3C65, PE; volume per test = lot dependent, final staining concentration = 5 µg ml^−1^) and PD-1 (BioLegend, NAT105-PE-Cy7; volume per test = 5, final staining concentration = 2.5 µg ml^−1^; T cell exhaustion and senescence). Flow cytometry was performed using BD FACSDIVA software (BD Biosciences) and FlowJo Single Cell Analysis Software v9.0 (FlowJo, LLC). For phenotypic characterization assays, gating-defined populations were established based on cell-mixed isotype controls for each individual marker. For DP characterization, T cell phenotypes were defined from the population of CAR^+^ T cells, while for PBMC characterization, T cell phenotypes were defined from CD3^+^ T cells. Cell viability was also evaluated via flow cytometry using a live/dead cell stain. Gating thresholds for live compared with dead cell populations were established based on fluorescence minus one controls.

To evaluate antigen-specific function (IFN-γ secretion) of ide-cel, DPs were thawed, washed and cultured in the presence of recombinant human BCMA. After 22–24 h, culture supernatants were collected, and the concentration of IFN-γ secretion was assessed via automated immunoassay (Protein Simple, Human IFNγ Single Plex).

Prior therapy washout was calculated for six common drug classes (HSCT, alkylating agents, anti-CD38 agents, corticosteroids, IMiDs and proteasome inhibitors) and was defined as the date of last known exposure until the date of apheresis for CAR T. The washout time was transformed as 1 per washout for patients with a recorded prior exposure; nonexposure was represented by a value of 0. A Spearman correlation was then used to test for a relationship between PBMC attributes and time-since-last-exposure for each drug class.

### Statistical analysis

The sample size planned for this study was based on clinical considerations and a standard dose-escalation design, as previously described^19^. Descriptive statistics include means with s.d. or medians with range for continuous variables; categorical variables were described by counts and percentages. Unless otherwise specified, missing data were not imputed. DOR, PFS and associated 95% CIs were estimated with the use of Kaplan–Meier methods. Censoring of data for PFS and response duration was based on US Food and Drug Administration censoring rules^32^. In the assessment of correlates of long-term responders with ide-cel in PBMCs and DPs, logistic regression analyses were performed adjusting for dose. Because of the exploratory nature of these studies, no adjustments for multiple comparisons were made and significance thresholds of ≤0.1 were used.

Analyses were performed with SAS software, version 9.4.

### Reporting summary

Further information on research design is available in the Nature Portfolio Reporting Summary linked to this article.

## Online content

Any methods, additional references, Nature Portfolio reporting summaries, source data, extended data, supplementary information, acknowledgements, peer review information; details of author contributions and competing interests; and statements of data and code availability are available at 10.1038/s41591-023-02496-0.

## Supplementary information

Supplementary Information Supplementary Tables 1 and 2 and Supplementary Figs. 1–4.

Reporting Summary

## Data Availability

De-identified and anonymized data will be made available within a secured portal to qualified researchers who submit an in-scope proposal approved by the Independent Review Committee. Proposals will be reviewed to ensure that there is adequate scientific rationale and methodology, a robust statistical analysis plan and a publication plan. Researchers should have relevant experience and demonstrate a plan to address any conflicts of interest, if applicable. Requests will be reviewed and processed by an independent committee; consequently, Bristol Myers Squibb cannot provide an estimated response time. For more information and to submit a data-sharing request, please visit https://www.bms.com/researchers-and-partners/independent-research/data-sharing-request-process.html
