# Supplementary material for: Idecabtagene vicleucel for relapsed and refractory multiple myeloma: post hoc 18-month follow-up of a phase 1 trial
Source: Nat Med. 2023 Aug 17;29(9):2286–94. doi: 10.1038/s41591-023-02496-0 (PMC10504071; doi:10.1038/s41591-023-02496-0)

nature medicine

Article https://doi.org/10.1038/s41591-023-02496-0

Idecabtagene vicleucel for relapsed and refractory multiple myeloma: post hoc 18-month follow-up of a phase 1 trial

In the format provided by the authors and unedited

Supplemental Material

Supplemental Table 1. Baseline characteristics and prior treatments in dose-escalation and dose-expansion cohorts

**Characteristics**	**Dose escalation (n = 21)**	**Dose expansion (n = 41)**
Age, median (range), years	57 (37-74)	63 (44-75)
Male, n (%)	13 (61.9)	26 (63.4)
Time since diagnosis, median (range), years	4.2 (1.4-15.9)	5.9 (0.8-35.7)
ECOG PS 0/1, %[a]		
0	8 (38.1)	8 (19.5)
1	11 (52.4)	33 (80.5)
High-risk cytogenetics, n (%)[b]	7 (33.3)	10 (24.4)
R-ISS III at baseline, n (%)	3 (14.3)	8 (19.5)
High tumor burden, n (%)[c]	10 (47.6)	17 (41.5)
Extramedullary disease, n (%)	4 (19.0)	19 (46.3)
Refractory to last prior therapy, n (%)	8 (38.1)	39 (95.1)
No. of prior regimens, median (range)	6 (3-15)	6 (3-18)
Prior ASCT, n (%)	21 (100)	36 (87.8)
Prior therapies, exposed / refractory, %		
Bortezomib	100 / 52.4	95.1 / 61.0
Carfilzomib	90.5 / 52.4	92.7 / 68.3
Ixazomib	23.8 / 23.8	31.7 / 24.4
Lenalidomide	100 / 71.4	100 / 73.2
Pomalidomide	90.5 / 61.9	95.1 / 87.8
Daratumumab	71.4 / 38.1	100 / 97.6
Isatuximab	14.3 / 14.3	4.9 / 4.9
Immunomodulatory and PI	100 / 71.4	100 / 85.4
Immunomodulatory, PI, and anti-CD38	81.0 / 42.9	100 / 82.9
Bridging therapy, n (%)	8 (38.1)	24 (58.5)

[a]Two patients in the dose-escalation group had ECOG PS 2. [b]del17p, t(4;14), and/or t(14;16). [c]≥ 50% CD138-positive cells or percentage of plasma cells in bone marrow biopsy. ASCT, autologous stem cell transplant; CAR, chimeric antigen receptor; ECOG PS, Eastern Cooperative Oncology Group performance status; PI, proteasome inhibitor; R-ISS, Revised International Staging System.

Supplemental Table 2. Tumor response in dose-escalation and dose-expansion cohorts

	150×10^6 cells		450×10^6 cells	
	Dose escalation (n = 6)	Dose expansion (n = 12)	Dose escalation (n = 9)	Dose expansion (n = 29)
Stringent complete response	4 (66.7)	2 (16.7)	4 (44.4)	8 (27.6)
Complete response	0	1 (8.3)	0	2 (6.9)
Very good partial response	0	0	4 (44.4)	12 (41.4)
Partial response	0	2 (16.7)	1 (11.1)	3 (10.3)
Overall response rate	4 (66.7)	5 (41.7)	9 (100)	25 (86.2)
≥ Very good partial response	4 (66.7)	3 (25.0)	8 (88.9)	22 (75.9)

Data presented as n (%).

Supplemental Figure 1. CONSORT diagram. [a]One patient who received 205 × 10^6 CAR+ T cells and 1 who received 305 × 10^6 are included under the 450 × 10^6 target dose. ide-cel, idecabtagene vicleucel; PD, progressive disease.

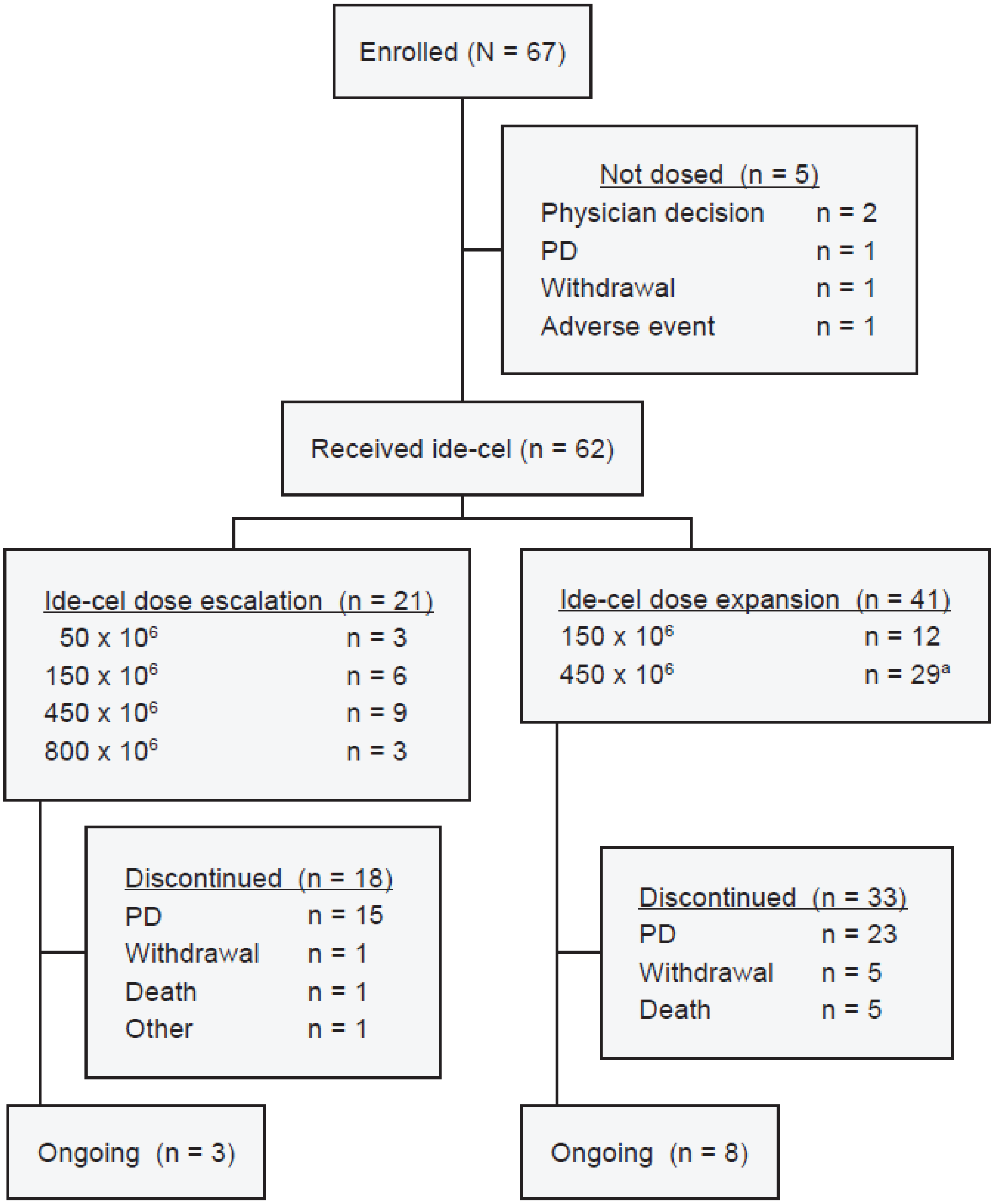

Supplemental Figure 2. Efficacy outcomes by target dose, including: a) best overall response, b) duration of response, c) progression-free survival, and e) overall survival.

CR, complete response; DOR, duration of response; ORR, overall response rate; PR, partial response; sCR, stringent complete response; VGPR, very good partial response.

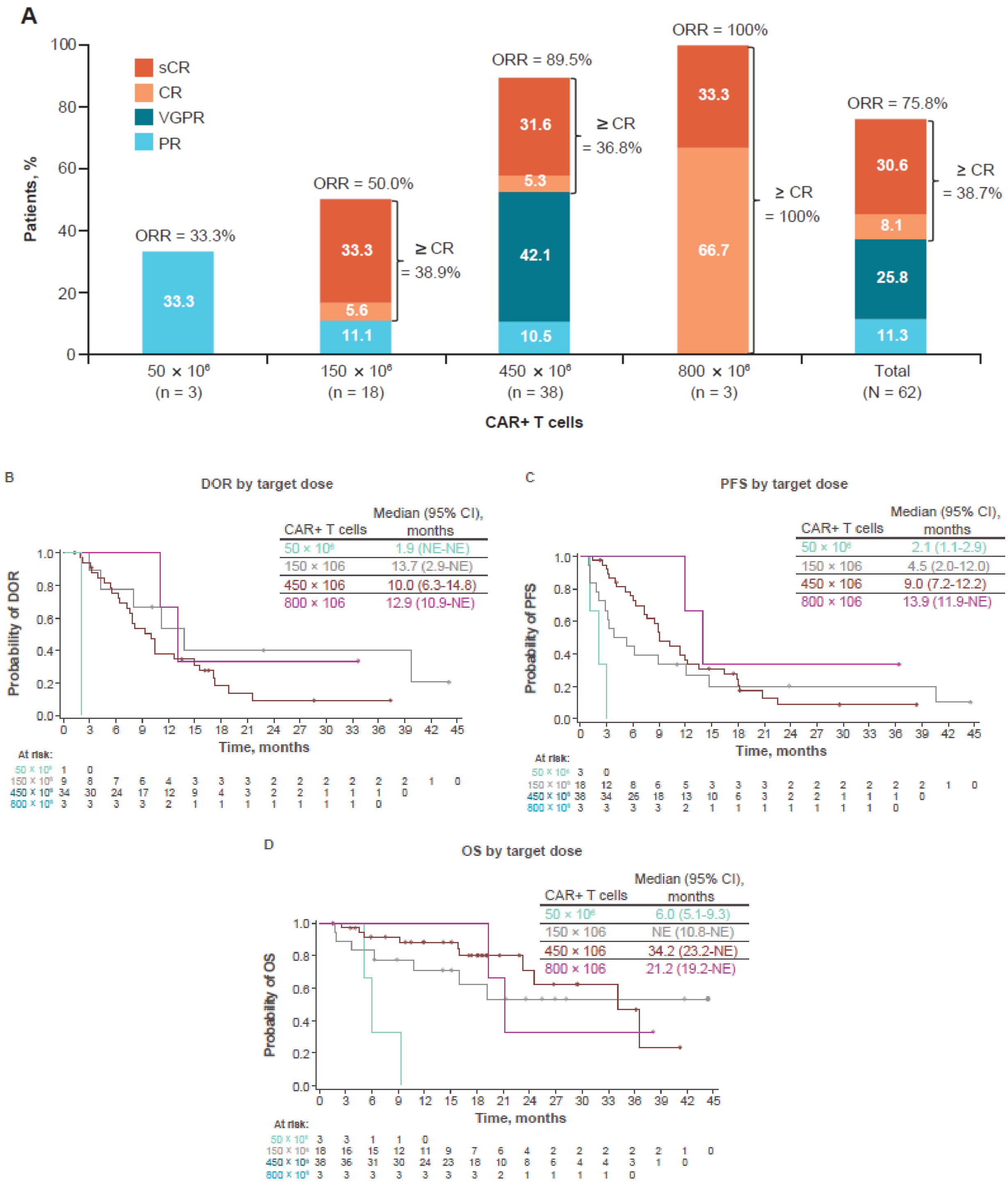

Supplemental Figure 3. DOR in patients treated with 150-450 × 10^6 CAR+ T cells by (Aa) age, (B) R-ISS, (C) extramedullary plasmacytoma, and (D) bridging therapy. DOR, duration of response; R-ISS, Revised International Staging System.

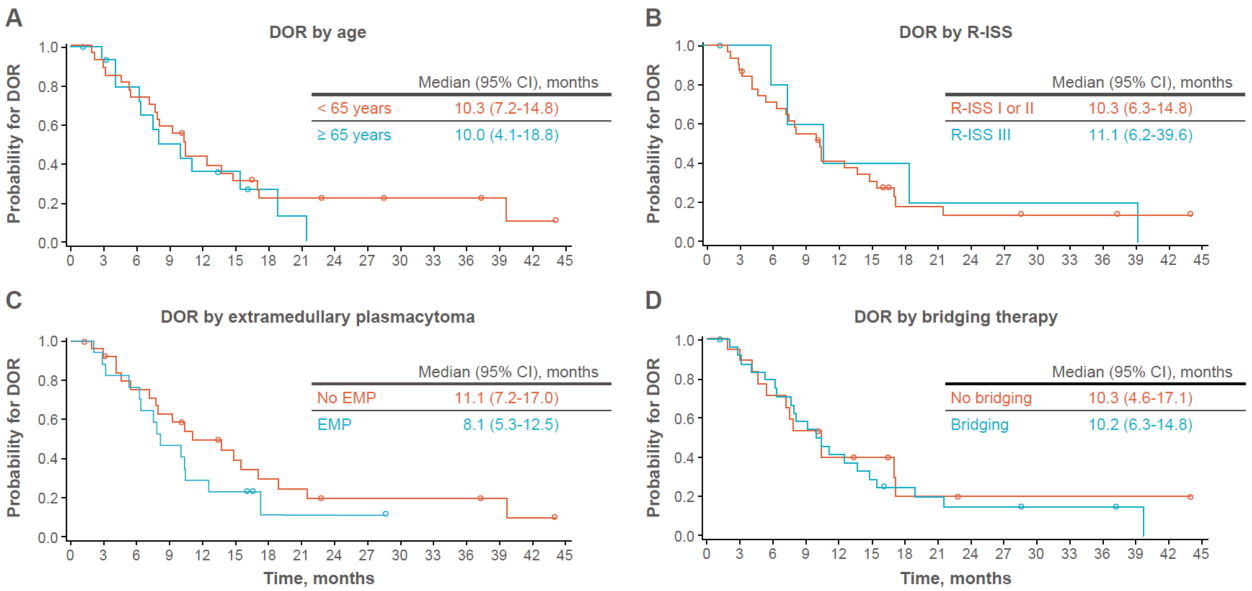

Supplemental Figure 4. Swimmer plots for patients with ≥ CR and MRD assessments. One patient who received 205 × 10^6 CAR+ T cells and 1 who received 305 × 10^6 are included under the 450 × 10^6 target dose. MRD assessments after PD not shown. Note that the end of each bar represents the earliest of the study discontinuation rate, cutoff date, or first PD date. The small black arrow indicates ongoing treatment response. AMT data not collected for all patients. AMT, anti-myeloma treatment; CR, complete response; Indet, indeterminate; MRD, minimal residual disease; PD, progressive disease; PR, partial response; sCR, stringent complete response; VGPR, very good partial response.

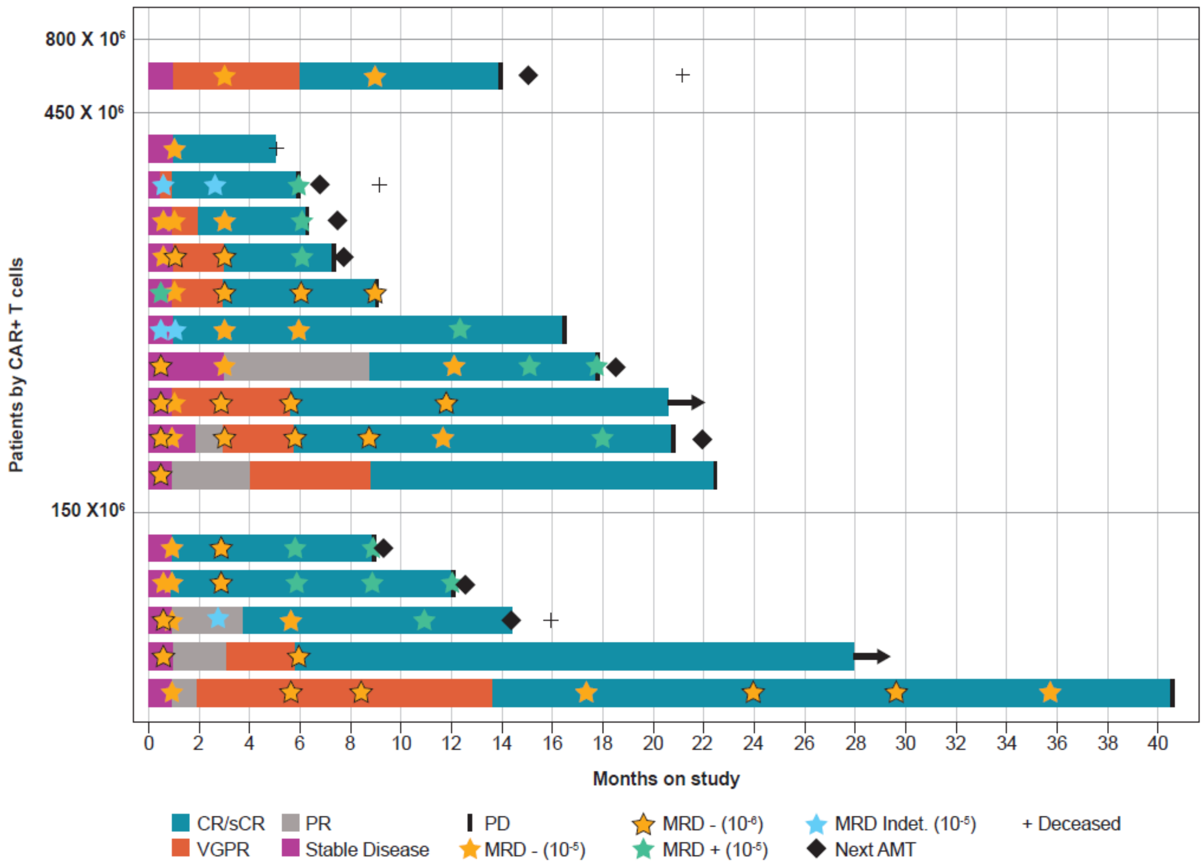

Supplement: Supplementary file 1 — Supplementary Tables 1 and 2 and Supplementary Figs. 1–4. [file 41591_2023_2496_MOESM1_ESM.pdf]
