# Supplementary material for: Idecabtagene vicleucel for relapsed and refractory multiple myeloma: post hoc 18-month follow-up of a phase 1 trial
Source: Nat Med. 2023 Aug 17;29(9):2286–94. doi: 10.1038/s41591-023-02496-0 (PMC10504071; doi:10.1038/s41591-023-02496-0)
Supplement: Supplementary file 2 — Reporting Summary [file 41591_2023_2496_MOESM2_ESM.pdf]

nature portfolio

Corresponding author(s): Yi Lin, MD

Last updated by author(s): Jun 28, 2023

Reporting Summary

Nature Portfolio wishes to improve the reproducibility of the work that we publish. This form provides structure for consistency and transparency in reporting. For further information on Nature Portfolio policies, see our Editorial Policies and the Editorial Policy Checklist.

Statistics

For all statistical analyses, confirm that the following items are present in the figure legend, table legend, main text, or Methods section.

n/a	Confirmed	
☐	☒	The exact sample size (*n*) for each experimental group/condition, given as a discrete number and unit of measurement
☐	☒	A statement on whether measurements were taken from distinct samples or whether the same sample was measured repeatedly
☐	☒	The statistical test(s) used AND whether they are one- or two-sided<br>*Only common tests should be described solely by name; describe more complex techniques in the Methods section.*
☐	☒	A description of all covariates tested
☐	☒	A description of any assumptions or corrections, such as tests of normality and adjustment for multiple comparisons
☐	☒	A full description of the statistical parameters including central tendency (e.g. means) or other basic estimates (e.g. regression coefficient) AND variation (e.g. standard deviation) or associated estimates of uncertainty (e.g. confidence intervals)
☐	☒	For null hypothesis testing, the test statistic (e.g. *F*, *t*, *r*) with confidence intervals, effect sizes, degrees of freedom and *P* value noted<br>*Give P values as exact values whenever suitable.*
☒	☐	For Bayesian analysis, information on the choice of priors and Markov chain Monte Carlo settings
☒	☐	For hierarchical and complex designs, identification of the appropriate level for tests and full reporting of outcomes
☒	☐	Estimates of effect sizes (e.g. Cohen's *d*, Pearson's *r*), indicating how they were calculated

Our web collection on statistics for biologists contains articles on many of the points above.

Software and code

Policy information about availability of computer code

Data collection	Flow cytometry was performed using BD FACSDIVA™ software (BD Biosciences) and FlowJo™ Single Cell Analysis Software v9.0 (FlowJo, LLC).
Data analysis	Analyses were performed with SAS software, version 9.4.

For manuscripts utilizing custom algorithms or software that are central to the research but not yet described in published literature, software must be made available to editors and reviewers. We strongly encourage code deposition in a community repository (e.g. GitHub). See the Nature Portfolio guidelines for submitting code & software for further information.

Data

Policy information about availability of data

All manuscripts must include a data availability statement. This statement should provide the following information, where applicable:

- Accession codes, unique identifiers, or web links for publicly available datasets
- A description of any restrictions on data availability
- For clinical datasets or third party data, please ensure that the statement adheres to our policy

De-identified and anonymized data will be made available within a secured portal to qualified researchers who submit an in-scope proposal approved by the Independent Review Committee. Proposals will be reviewed to ensure that there is adequate scientific rationale and methodology, a robust statistical analysis plan, and a publication plan. Researchers should have relevant experience and demonstrate a plan to address any conflicts of interest, if applicable. For more information and to submit a data sharing request, please visit https://www.bms.com/researchers-and-partners/independent-research/data-sharing-request-process.html

Research involving human participants, their data, or biological material

Policy information about studies with human participants or human data. See also policy information about sex, gender (identity/presentation), and sexual orientation and race, ethnicity and racism.

Reporting on sex and gender	In this Phase 1 trial, adverse events and response rates were analyzed by sex/gender. Data stratified by sex/gender were not collected in the post-hoc analysis.
Reporting on race, ethnicity, or other socially relevant groupings	Data based on race and ethnicity were not collected in the post-hoc analysis.
Population characteristics	Patients had a median age of 61 years; 62.9% of patients were male and 37.1% female; 25.8% of patients had an ECOG PS of 0 and 71% of patients had and ECOG PS of 1.
Recruitment	Patients were recruited by investigators through enrollment at select sites based on prespecified inclusion/exclusion criteria.
Ethics oversight	The protocol was reviewed and approved at each study site. Principles were outlined in the Declaration of Helsinki. Institutional review boards approved the protocol at each study center, and each patient provided written informed consent.

Note that full information on the approval of the study protocol must also be provided in the manuscript.

Field-specific reporting

Please select the one below that is the best fit for your research. If you are not sure, read the appropriate sections before making your selection.

☒ Life sciences ☐ Behavioural & social sciences ☐ Ecological, evolutionary & environmental sciences

For a reference copy of the document with all sections, see nature.com/documents/nr-reporting-summary-flat.pdf

Life sciences study design

All studies must disclose on these points even when the disclosure is negative.

Sample size

The sample size was based on clinical considerations and a standard dose-escalation design, as previously described (Raje et al, NEJM 2019; 380:1726-1737).

Data exclusions

From the protocol:
1. Treatment with the following therapies within the specified time period:
    a. Any prior systemic therapy for MM within 14 days prior to scheduled protocol-required leukapheresis
    b. Investigational cellular therapies within 8 weeks prior to the start of lymphodepletion.
2. Subjects with known central nervous system (CNS) disease. History or presence of clinically relevant CNS pathology such as epilepsy, seizure, paresis, aphasia, stroke, subarachnoid hemorrhage or CNS bleed, severe brain injuries, dementia, Parkinson's disease, cerebellar disease, organic brain syndrome, or psychosis (Note: this criterion does not apply to subjects undergoing retreatment unless Grade 4 neurotoxicity was observed following prior treatment with bb2121)
3. Inadequate hepatic function defined by aspartate aminotransferase (AST) and/or alanine aminotransferase (ALT) >2.5 × upper limit of normal (ULN) and direct bilirubin >1.5 × ULN
4. Inadequate renal function defined by creatinine clearance ≤45 ml/min using the Cockcroft-Gault formula
5. International ratio (INR) or partial thromboplastin time (PTT) >1.5 × ULN
6. Inadequate bone marrow function defined by absolute neutrophil count (ANC) <1000 cells/mm3 in the absence of growth factor support (Neupogen within 7 days or Neulasta within 14 days) and platelet count <50,000 cells/mm3, in the absence of transfusion support (platelet transfusion within 7 days)
7. Left ventricular ejection fraction <50%
8. Ongoing treatment with chronic immunosuppressants (e.g., cyclosporine or systemic steroids at any dose). Intermittent topical, inhaled, or intranasal corticosteroids are allowed
9. Presence of active infection within 72 hours prior to leukapheresis or lymphodepletion; subjects with ongoing use of prophylactic antibiotics, antifungals, or antivirals are eligible as long as there is no evidence of active infection
10. Previous history of an allogeneic bone marrow transplantation or treatment with any gene therapy-based therapeutic for cancer
11. Significant co-morbid condition or disease which in the judgment of the Investigator would place the subject at undue risk or interfere with the study; examples include, but are not limited to, cirrhotic liver disease, sepsis, recent significant traumatic injury, and other conditions
12. Known human immunodeficiency virus (HIV) positivity
13. Subjects with a history of class III or IV congestive heart failure or non-ischemic cardiomyopathy, unstable angina, myocardial infarction, or ventricular arrhythmia requiring medication or mechanical control within the previous 6 months
14. Subjects with second malignancies in addition to myeloma, if the second malignancy has required therapy in the last 3 years or is not in complete remission; exceptions to this criterion include successfully treated non-metastatic basal cell or squamous cell skin carcinoma, or prostate cancer that does not require therapy
15. Subjects who have had a venous thromboembolic event (e.g., pulmonary embolism or deep vein thrombosis) requiring anticoagulation and who meet any of the following criteria:
    15a. Requires ongoing therapeutic anticoagulation
    15b. Have had a Grade 2, 3, or 4 hemorrhage in the last 30 days
    15c. symptoms from their venous thromboembolic event (e.g. continued dyspnea or oxygen requirement)

15d. NOTE: Subjects who have had a venous thromboembolic event but do not meet any of the above 3 criteria are eligible for participation
16. Subjects who have plasma cell leukemia or clinically significant amyloidosis
17. Pregnant or lactating women

Replication	Given limited patient sample availability, there were no attempts at replication with respect to peripheral blood mononuclear cell (PBMC) and drug product (DP) characterization.
Randomization	There was no randomization in this Phase 1 clinical study. No formal statistical analysis that adjusted for possible covariate effects was planned nor conducted.
Blinding	Since this was a single-arm study, all subjects received ide-cel infusion. Teatment assignment did not require randomization, blinding, or stratification.

Reporting for specific materials, systems and methods

We require information from authors about some types of materials, experimental systems and methods used in many studies. Here, indicate whether each material, system or method listed is relevant to your study. If you are not sure if a list item applies to your research, read the appropriate section before selecting a response.

Materials & experimental systems

n/a	Involved in the study	
☐	☒	Antibodies
☒	☐	Eukaryotic cell lines
☒	☐	Palaeontology and archaeology
☒	☐	Animals and other organisms
☐	☒	Clinical data
☒	☐	Dual use research of concern
☒	☐	Plants

Methods

n/a	Involved in the study	
☒	☐	ChIP-seq
☐	☒	Flow cytometry
☒	☐	MRI-based neuroimaging

Antibodies

Antibodies used

1. CD45RA: BD Biosciences, HI100-BV605, Catalog No. 562886
2. CCR7: BD Biosciences, 150503-BV421, Catalog No. 562555
3. CD28: ThermoFisher, CD28.2-PE eFlour610, Catalog No. 61-0289-42
4. CD27: BD Biosciences, L128-FITC, Catalog No. 340424
5. CD57: BD Biosciences, NK-1-BB515, Catalog No. 565285
6. TIM-3: ThermoFisher, F38-2E2-Super Bright 600, Catalog No. 63-3109-42
7. LAG-3: BioLegend, 11C3C65, PE, Catalog No. 369306
8. PD-1: BioLegend, NAT105-PE-Cy7, Catalog No. 367414

Validation

For CD45RA (BD Biosciences, HI-100, BV605); CCR7 (BD Biosciences, 150503-BV421); CD27 (BD Biosciences, L128-FITC); and CD57 (BD Biosciences, NK-1-BB515): The production process underwent stringent testing and validation to assure that it generates a high-quality conjugate with consistent performance and specific binding activity. However, verification testing has not been performed on all conjugate lots.

For CD28 (ThermoFisher, CD28.2-PE Fluor 610): This antibody has been verified by Cell treatment and Relative expression to confirm specificity to CD28.

For TIM-3 (ThermoFisher, F38-2E2-Super Bright 600): This antibody has been verified by cell Treatment to confirm specificity to TIM-3.

For LAG-3 (Biolegend, 11C3C65, PE): Each lot of this antibody is quality control tested by immunofluorescent staining with flow cytometric analysis. For flow cytometric staining, the suggested use of this reagent is 5 µl per million cells in 100 µl staining volume or 5 µl per 100 µl of whole blood.

For PD-1 (Biolegend, NAT105-PE-Cy7): Each lot of this antibody is quality control tested by immunofluorescent staining with flow cytometric analysis. For flow cytometric staining, the suggested use of this reagent is 5 µl per million cells in 100 µl staining volume or 5 µl per 100 µl of whole blood.

Clinical data

Policy information about clinical studies
All manuscripts should comply with the ICMJE guidelines for publication of clinical research and a completed CONSORT checklist must be included with all submissions.

Clinical trial registration: NCT02658929

Study protocol	Protocol will be uploaded to the journal upon submission and avaialble online after the manscript is accepted for publication.
Data collection	Data were collected at the following US-based hospitals, cancer centers, and oncology research organizations:<br>1. Stanford Cancer Center, Stanford, California<br>2. National Cancer Institute, Bethesda, Maryland<br>3. Massachusetts General Hospital, Boston, Massachusetts<br>4. Boston, Massachusetts<br>5. Dana Farber Cancer Institute, Boston, Massachusetts<br>6. Mayo Clinic, Rochester, Minnesota, US<br>7. Hackensack, New Jersey<br>8. Mt. Sinai Medical Center, Division of Hematology/Oncology, New York, New York,<br>9. Sarah Cannon Research Institute, Nashville, Tennessee
Outcomes	The primary end point was safety. Severity was graded according to National Cancer Institute Common Terminology Criteria for Adverse Events, version 4.03. Cytokine release syndrome was defined and graded according to published criteria. Neurotoxicity was reported ≤ 8 weeks after infusion as a grouped term (comprising preferred terms including, but not limited to, bradyphrenia, brain edema, confusional state, hallucination, insomnia, lethargy, memory impairment, neurotoxicity, nystagmus, somnolence, and tremor).<br><br>Secondary end points were ORR, CR rate, VGPR, and PR according to International Myeloma Working Group (IMWG) criteria.<br><br>Exploratory end points included the following: Evaluation of minimal residual disease by next-generation sequencing with the use of a minimum cutoff of 10–4 nucleated cells (clonoSEQ, Adaptive Biotechnologies) at specified time points, independent of response status; PFS, which was calculated as the time (in months) from the date of ide-cel infusion to the first date of documented PD or death due to any cause, whichever occurred earlier; and OS, which was calculated as the time from the ide-cel infusion date to the date of death due to any cause.<br><br>Additional exploratory endpoints included detection and quantification of ide-cel in blood, bone marrow, and/or tumor tissue over time and detection and quantification of circulating soluble BCMA over time.

Flow Cytometry

Plots

Confirm that:

- [ ] The axis labels state the marker and fluorochrome used (e.g. CD4-FITC).
- [ ] The axis scales are clearly visible. Include numbers along axes only for bottom left plot of group (a 'group' is an analysis of identical markers).
- [ ] All plots are contour plots with outliers or pseudocolor plots.
- [ ] A numerical value for number of cells or percentage (with statistics) is provided.

Methodology

Sample preparation	Cellular kinetics (peripheral blood and bone marrow) were evaluated as previously described (Raje et al, NEJM 2019; 380:1726-1737). Briefly, T-cell phenotyping was performed on both PBMC starting material from apheresis and drug product (DP) to evaluate T-cell memory subsets, as well as phenotypes associated with senescence and T-cell dysfunction.<br><br>Cellular kinetics were evaluated by qPCR and results were analyzed for associations with response and DOR in patients with matched day 14 visit samples in CD3+ cells sorted by flow cytometry from peripheral blood and in whole bone marrow aspirate.
Instrument	Samples were acquired on a BD FACSCanto II™ cell analyzer (BD Biosciences).
Software	BD FACSDIVA™ Software (BD Biosciences) or FlowJo Single Cell Analysis Software v9.0 (FlowJo, LLC).
Cell population abundance	The intensity of the protein markers was binarized using the silhouette distance method based on a reference sample that was spiked into each sample. The primary cell types in the spiked-in cells serve as positive and negative references for each protein, from which the threshold to establish protein positivity in the subject cells is computed. Differentially enriched proteins or cell types were identified using a generalized linear model (GLM) with a quasibinomial distribution adjusted for dose. Proteins with positive coefficients from the GLM model depict an enrichment in patients with long-term response.
Gating strategy	Not applicable.

- [ ] Tick this box to confirm that a figure exemplifying the gating strategy is provided in the Supplementary Information.
